# Surface-Modified Electrospun Glass Nanofibers from Silane Treatment and Their Use for High-Performance Epoxy-Based Nanocomposite Materials

**DOI:** 10.3390/ma16206817

**Published:** 2023-10-23

**Authors:** Abhijeet Mali, Philip Agbo, Shobha Mantripragada, Lifeng Zhang

**Affiliations:** Department of Nanoengineering, Joint School of Nanoscience and Nanoengineering, North Carolina A&T State University, 2907 E Gate City Blvd, Greensboro, NC 27401, USA

**Keywords:** electrospinning, glass nanofiber, epoxy nanocomposite, surface modification, silane coupling agent

## Abstract

As a new and promising reinforcing filler, electrospun glass nanofibers (EGNFs) have attracted attention in the field of polymer composite materials. However, the reinforcing effectiveness of surface-modified EGNFs using different silane coupling agents in epoxy resin is still not quite clear. In this research, a series of silane coupling agents with increasing chain lengths in the order of methyl trimethoxysilane (MTMS), (3-aminopropyl) triethoxysilane (APTES), (3-glycidyloxypropyl) trimethoxysilane (GPTMS), and dual silane coupling agent APTES–GPTMS were employed to carry out surface treatment on the EGNFs. The pristine and silane functionalized EGNFs were then incorporated into epoxy resin as reinforcing fillers at low loading levels, i.e., 0.25 wt.%, 0.5 wt.%, and 1 wt.%, and the mechanical properties of the resultant epoxy nanocomposites, including strength, stiffness, ductility, and toughness, were evaluated. A commercial product of glass nanoparticles (GNPs) was used as a control to compare the reinforcing effectiveness of the EGNFs and the GNPs. This study revealed that the EGNFs could provide significant reinforcing and toughening effects at ultra-low loading (0.25 wt.%) in epoxy nanocomposite materials. Furthermore, surface modification of the EGNFs with silane coupling agents with long chain lengths, e.g., by using dual silane coupling agents, APTES–GPTMS, could enhance the interfacial bonding between the EGNFs and the epoxy matrix and further increase the mechanical performance of the EGNF-reinforced epoxy nanocomposite materials. Through this research, we realized epoxy nanocomposite materials with much-improved mechanical properties, i.e., 37%, 24%, 18%, 57% improvement in strength, stiffness, ductility, and toughness, respectively, with respect to those of the cured neat epoxy material with an ultra-low loading (0.25 wt.%) of APTES–GPTMS–EGNFs. Our research paves the road for developing lighter and stronger epoxy nanocomposite materials with EGNFs.

## 1. Introduction

Nano-scaled fillers have gained substantial attention in the field of polymer matrix composites (PMCs) due to their notable ability to improve the mechanical properties of PMCs [1,2,3]. In recent years, there have been increasing research efforts geared toward electrospun nanofiber-reinforced composite materials [4]. Among all electrospun nanofibers that are currently involved in reinforcing PMCs, polymer-based nanofibers have, so far, received the most consideration because they were developed first from electrospinning and have matured, relatively, for applications. Glass and ceramic nanofibers are examples of non-polymer nanofibers that have been developed recently through electrospinning a spin dope containing glass/ceramic precursor materials followed by sol–gel processing and subsequent pyrolysis at elevated temperatures with aims of exploring their energy, electronic, and catalytic utilizations [5,6]. Due to readily available precursors, e.g., tetraethyl orthosilicate (TEOS), chemical inertness, thermal stability, low thermal conductivity, and biocompatibility, electrospun glass (SiO_2_) nanofibers (EGNFs) have been used for energy, industrial catalysis, insulation, separation, and biomedical applications [7,8,9,10,11]. A noteworthy fact about EGNFs is that they may possess outstanding tensile strength and modulus like those of bulk glass [12] and can, therefore, be used as reinforcing fillers to make high-performance nanofiber-reinforced PMCs [4]. EGNFs have been employed as reinforcing agents in PMCs, including both polymer matrix reinforcement and composite laminate reinforcement [13,14]. In addition to their reinforcing effect, EGNFs could also be functionalized with alkaline earth aluminates or lanthanide-doped aluminate nanoparticles to develop UV-responsive and photochromic smart materials [15,16,17].

Epoxy resins are a popular class of thermoset prepolymers, and they are extensively used as matrices in fiber-reinforced polymer composite materials, coatings, and adhesives in a wide range of industries, such as aerospace, construction, transportation, and electronics, due to their excellent physical properties after curing [18,19,20]. In our previous research, EGNFs demonstrated great potential to reinforce epoxy-based polymer nanocomposite materials at relatively low loading levels [21]. However, the agglomeration of EGNFs becomes more significant at high loading levels, due to their ultra-high surface area and surface energy, insufficient wetting of EGNFs (inorganic) by epoxy resin (organic), and interaction between silanol groups on the surface of EGNFs. Thus, further investigation is needed to clarify the reinforcing effectiveness of EGNFs in epoxy resin, especially when we perform surface modification to EGNFs. There have been no previous reports on the effects of the surface-grafted silane molecular chain length of EGNFs on the mechanical properties of the resultant epoxy-based nanocomposite materials after surface modification of EGNFs using different silane coupling agents.

In this study, we addressed the abovementioned research gap and aimed to comprehend the role of grafted silane molecular chain length as a contributing factor in the mechanical performance of the resultant epoxy-based nanocomposite materials. We performed surface modification to the EGNFs using silane coupling agents with increasing chain lengths, including methyl trimethoxysilane (MTMS), (3-aminopropyl) triethoxysilane (APTES), (3-glycidyloxypropyl) trimethoxysilane (GPTMS), and dual silane APTES–GPTMS, and evaluated the performance of these surface-modified EGNFs in reinforcing epoxy resin, including the mechanical properties of strength, stiffness, ductility, and toughness at low loading levels (0.25–1 wt.%). A commercial product of glass nanoparticles (GNPs) was used as a control to compare the reinforcing effects of EGNFs and GNPs in the epoxy matrix. Our research findings provide in-depth information to understand the reinforcing effect of EGNFs in epoxy-based nanocomposite materials, as well as directions for further enhancing their reinforcing effect in future EGNF-reinforced high-performance epoxy nanocomposite materials.

## 2. Experimental

### 2.1. Materials

The epoxy resin (EPON 862) and corresponding curing agent of EPIKURE W were purchased from Miller Stephenson (Danbury, CT, USA). Tetraethyl orthosilicate (TEOS, 98%) was purchased from Acros Organics (Waltham, MA, USA). Polyvinylpyrrolidone (PVP, Mw = 1,300,000), N, N-dimethylformamide (DMF, 99%), dimethyl sulfoxide (DMSO, 98%), methyl trimethoxysilane (MTMS), (3-aminopropyl) triethoxysilane (APTES), (3-glycidyloxypropyl) trimethoxysilane (GPTMS), glass nanoparticles (GNPs, i.e., SiO_2_ nanoparticles, average diameter 20 nm, and BET surface area 200 m^2^/g) were purchased from Sigma-Aldrich (St. Louis, MO, USA). Ethanol and anhydrous acetone were purchased from Fisher Scientific (Waltham, MA, USA). All the chemicals were used as received without further purification.

### 2.2. Preparation of Electrospun Glass Nanofibers (EGNFs)

A comprehensive study of the synthesis of EGNFs was reported in our previous publication [22]. Specifically, in this research, a spin dope consisting of 13 wt.% TEOS and 13 wt.% PVP in a DMF/DMSO (2/1, wt./wt.) mixture solvent was used to electrospin glass precursor nanofibers. The electrospinning was carried out with a feeding ratio of 1 mL/h at a constant voltage of 15 kV and a distance of 20 cm between the tip of needle and the collector in a Spinbox electrospinning unit (Nanoscience Instruments, Phoenix, AZ, USA). The obtained glass precursor nanofibers in the form of a non-woven mat were pyrolyzed in a Carbolite (Derbyshire, UK) furnace at 350 °C for 3 h and 800 °C for 6 h with a ramp heating rate of 10 °C/min and a constant air flow to obtain the final product of the EGNFs.

### 2.3. Surface Modification

**Figure 1:**
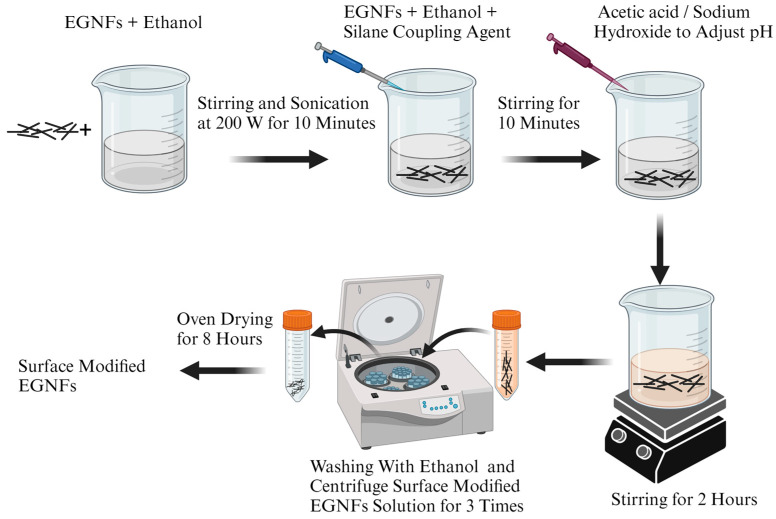
Schematic diagram of EGNF surface-modification process.

Three different silane coupling agents with increasing chain lengths, including MTMS, APTES, and GPTMS, were employed to modify the surface of the prepared EGNFs. In addition, a combination of silane coupling agents, i.e., APTES+GPTMS, was also employed to functionalize the EGNFs with even longer chain lengths. During the surface modification process (Figure 1), the EGNFs were first dispersed in ethanol at a weight fraction of 1.5 wt.%. The resultant suspension was subjected to a vigorous ultrasonication via a 500 W ultrasonic probe sonicator (QSONICA, Newtown, CT, USA) at 40% power for 5 min to achieve a uniform dispersion of the EGNFs in ethanol. Subsequently, a corresponding silane coupling agent was added into the EGNF/ethanol dispersion at a concentration of 2 wt.%, followed by stirring at 600 rpm for 10 min. The pH of the reaction system was maintained at 4–5 by the addition of acetic acid for the APTES and MTMS, and at 10–11 by using 0.1 M NaOH solution for the GPTMS. The reaction of the EGNF surface modification was carried out for 2 h with continuous stirring at 600 rpm. Subsequently, the surface-functionalized EGNFs (MTMS–EGNFs, APTES–EGNFs, and GPTMS–EGNFs) were acquired by centrifugation and thoroughly washed three times with ethanol to remove the physically adsorbed silane molecules. To surface-modify the EGNFs with APTES+GPTMS, the EGNFs were first surface-modified with APTES and the obtained APTES–EGNFs were used as the starting material for further GPTMS modification, following the same procedure as mentioned above to obtain APTES–GPTMS-functionalized EGNFs (APTES–GPTMS–EGNFs). The commercial GNPs were also surface-modified using the same procedure to obtain MTMS–GNPs, APTES–GNPs, GPTMS–GNPs, and APTES–GPTMS–GNPs for comparison with the surface-modified EGNFs. All the surface-modified EGNFs and GNPs were dried in a vacuum oven at 60 °C for 8 h before further use.

### 2.4. Fabrication of EGNF-Reinforced Epoxy Nanocomposite

Pristine and surface-modified EGNFs were employed as reinforcing agents to make epoxy nanocomposites at loadings of 0.25 wt.%, 0.5 wt.%, and 1 wt.%, respectively. Each type of EGNFs was first mixed with acetone and sonicated for 5 min at 200 W to disintegrate EGNF agglomerates and homogenize them. Simultaneously, EPON 862 was heated and degassed in an oven under vacuum (27 mmHg) at 60 °C for 10 min. Then, the EGNF-acetone dispersion was mixed with the epoxy resin upon stirring at 600 rpm and 60 °C for 2 h to homogenously distribute the EGNFs in the epoxy resin and, simultaneously, to remove acetone. Subsequently, the epoxy resin with the dispersed EGNFs was further sonicated for another 10 min at 200 W to improve the distribution of the EGNFs in the epoxy matrix. Afterward, the curing agent (EPIKURE W) was added into the system at 26.4 wt.% of epoxy, and the mixture was further stirred at 600 rpm and 60 °C for 10 min to obtain a uniform mixture. The mixture was next degassed under a vacuum at 60 °C for 10 min to remove air bubbles. After being degassed, the epoxy resin system was poured into a 5 in. × 5 in. mold and cured at 350 °F for 4 h. Pristine and surface-modified GNPs were also used to fabricate the GNP-reinforced epoxy nanocomposites at loadings of 0.25 wt.%, 0.5 wt.%, 1 wt.%, 1.5 wt.%, and 2 wt.%, following the same procedure as described for the EGNFs, along with the neat epoxy (without any nanofillers), for comparison purpose.

### 2.5. Characterization

A Zeiss (White Plains, NY, USA) Auriga Crossbeam FIB field emission scanning electron microscope (FESEM) was used to examine morphology of the EGNFs and the GNPs, and fracture surfaces of the epoxy nanocomposites. Prior to SEM, all the samples were sputter coated with gold–palladium in a thickness of 7 nm to avoid charge accumulation. The average fiber diameter of the respective nanofiber sample was obtained by measuring the diameters of at least 30 randomly selected nanofibers in corresponding SEM images, using Image J software (NIH, Bethesda, MD, USA). A Varian (Palo Alto, CA, USA) 670 FTIR spectrometer was used to confirm the surface modification of the EGNFs and the GNPs with various silane coupling agents over the wavenumber range of 4000–500 cm^−1^. Dynamic light scattering (DLS—Malvern (Malvern, UK) Zetasizer Nano Range) was used to determine the zeta potential and the hydrodynamic diameter of all the EGNF and GNP samples. For each sample, specifically, the nanofiller was mixed in DI water with a weight ratio of 1:10,000 and sonicated for 5 min for uniform dispersion. 1 mL of liquid from the prepared mixture was then used for the DLS measurement. Dog-bone-shaped specimens were cut from the cured epoxy nanocomposite panels, using a water jet cutting machine (Flow (Kent, WA, USA)-MACH 2). The tensile test of all the cured epoxy nanocomposite materials was carried out using a universal testing machine (UTM Instron (Norwood, MA, USA) 3384) as per ASTM D638-Type IV, at room temperature. Five specimens of each sample were prepared and tested. The average measurement values and the standard deviations were calculated. The thermal stability test of the epoxy nanocomposites was performed by using a thermogravimetric analyzer (Q500 TGA, TA Instruments (New Castle, DE, USA)). The TGA analysis was carried out by heating a sample of ~10 mg, from room temperature to 800 °C, at a heat rate of 10 °C/min in a nitrogen environment, with a flow rate of 50 mL/min.

## 3. Results and Discussion

### 3.1. Morphology

**Figure 2:**
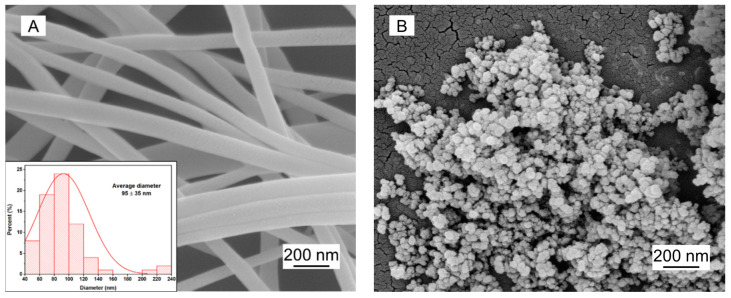
SEM images of EGNFs (**A**) and commercial GNPs (**B**). The inset of (**A**) shows the size distribution of EGNFs.

The EGNFs were collected in the form of non-woven mat and exhibited a good cylindrical fiber shape (Figure 2A). The diameters of the EGNFs varied from a minimum size of 50 nm to a maximum size of 240 nm, with an average diameter of 95 ± 35 nm. The commercial GNPs exhibited a spherical shape, with an average size of 20 nm according to the product specifications. The agglomeration of GNPs was observed, and some GNP agglomerates could even fall into the micrometer range.

### 3.2. Reinforcing Effect of Pristine EGNFs

The mechanical properties, including tensile strength, Young’s modulus, elongation at break, and work of fracture, of the epoxy nanocomposites reinforced by pristine EGNFs at loading levels of 0.25 wt.%, 0.5 wt.%, and 1 wt.%, respectively, were evaluated by tensile test. For comparison, the counterparts with the pristine GNPs at loading levels of 0.25 wt.%, 0.5 wt.%, 1 wt.%, 1.5 wt.% and 2 wt.%, respectively, were also investigated. The neat epoxy resin with only the hardener was cured and tested as a control.

**Figure 3:**
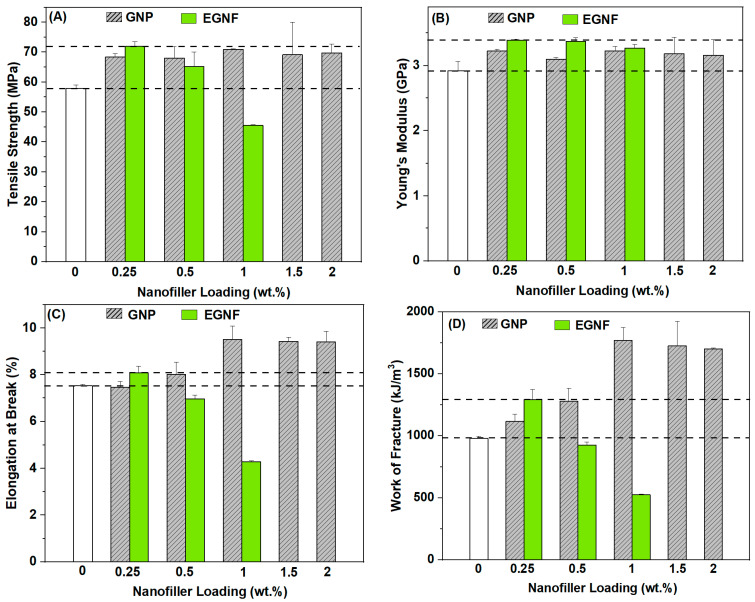
Tensile strength (**A**), Young’s modulus (**B**), elongation at break (**C**), and work of fracture (**D**) of neat epoxy (control sample) and reinforced epoxy nanocomposites with pristine EGNFs and GNPs.

Tensile strength indicates the maximum stress that a material can withstand prior to failure. The inclusion of nanofillers, both EGNFs and GNPs, resulted in significant improvements in the strength of epoxy nanocomposites, except at 1 wt.% of the EGNFs (Figure 3A). Among all the GNP-reinforced epoxy nanocomposites, 1 wt.% loading resulted in the maximum strength improvement of ~22%, compared to that of the neat epoxy at 57.9 MPa. With further increase of GNP loading, the strength of the epoxy nanocomposite started to decrease. The incorporation of EGNFs at 0.25 wt.% resulted in the greatest strength improvement among all the studied epoxy nanocomposites, i.e., 72 MPa, which was ~25% more than that of the neat epoxy. The increase in EGNF loading significantly degraded the strength of the EGNF-reinforced epoxy nanocomposites and the corresponding strengths were lower than those of the respective GNP counterparts. At 1 wt.% EGNF loading, the epoxy nanocomposite showed the poorest tensile strength among all the studied nanocomposite samples. It is well known that the agglomeration of nanofillers is a major reason for the poor mechanical properties of polymer nanocomposites. The much-reduced strength of EGNF-reinforced epoxy nanocomposites at higher loading could be attributed to the agglomeration of EGNFs. The observations indicated greater agglomeration of the EGNFs than GNPs at the higher loading levels (0.5 wt.% and 1 wt. %). The agglomeration of the EGNFs became seriously bad at 1 wt.% loading, which led to a ~21% strength reduction, compared to that of the neat epoxy. In contrast with the GNPs, the EGNFs could benefit from a “bridging” mechanism [21]. This is why the EGNFs showed a greater reinforcing effect than the GNPs at the 0.25 wt.% loading level, at which EGNF agglomeration is low.

Young’s modulus indicates the stiffness of a material. Incorporation of the glass nanofillers showed a positive effect on stiffness of the resultant epoxy nanocomposites (Figure 3B). All the studied glass nanofiller-reinforced epoxy nanocomposites showed improvement in stiffness, due to the large modulus of SiO_2_. Compared to the modulus of the neat epoxy (2.92 GPa), the moduli of the GNP-reinforced epoxy nanocomposites were observed in the range of 3.10–3.22 GPa (an improvement of up to 10%), while the moduli of the EGNF-reinforced epoxy nanocomposites were observed in the range of 3.27–3.39 GPa (an improvement of up to 16%).

Elongation at break is an important parameter for depicting the rupture behavior of a composite material, and it denotes the ductility of the material to undergo significant deformation before its failure [23]. The neat epoxy exhibited 7.5% of elongation at break (Figure 3C). Compared to the neat epoxy, the incorporation of GNPs showed almost no influence on the elongation at break at the loading of 0.25 wt.%, but improved it by up to 26.7% with higher loadings. At 0.25 wt.% loading level, the elongation at break of the EGNF-reinforced epoxy nanocomposite increased by ~7% with respect to that of the neat epoxy and outperformed the GNP counterpart. At higher loading levels, the EGNF-reinforced epoxy nanocomposites showed lower elongation at break than that of the GNP counterparts and the neat epoxy. Notably, we observed that the epoxy nanocomposite with 1 wt.% EGNF loading exhibited a ~43% drastic decrement in elongation at break. The improvement in elongation at break (ductility) of the EGNF- and GNP-reinforced epoxy nanocomposites could be ascribed to matrix shear banding [21]. The deteriorated ductility of epoxy nanocomposites with higher loadings of EGNFs could be caused by the severe EGNF agglomeration.

Work of fracture (WOF) is a parameter that characterizes the toughness of a material, and it denotes the energy absorbed by the specimen before its failure. The WOF is achieved by the area under the tensile stress–strain curve. This is a direct and efficient method for assessing the static toughness of materials, and it has gained widespread acceptance among researchers in the field of polymer composites [24,25]. Compared to the WOF of neat epoxy (977 kJ/m^3^, Figure 3D), the incorporation of GNPs provided an increment in WOF of the resultant epoxy nanocomposites over all the loading levels that were studied, and the maximum WOF improvement of ~81% was observed at 1 wt.% GNP loading. At 0.25 wt.% loading, the EGNF-reinforced epoxy nanocomposite showed a notable improvement of ~32% in WOF with respect to that of the neat epoxy and outperformed the GNP counterpart. However, the WOF of the epoxy nanocomposites decreased by 5% and 46%, respectively, at 0.5 wt.% and 1 wt.% EGNF loadings. As pointed out in our previous research [21], epoxy nanocomposites with GNPs and EGNFs could benefit from matrix shear banding and nanofiller debonding, while epoxy nanocomposites with EGNFs could further benefit from crack deflection and crack bridging. With the increase in EGNF loading, due to increasingly severe EGNF agglomeration, the toughening effect of EGNFs in the epoxy nanocomposites was totally lost. At 0.25 wt.% loading, the EGNFs outperformed the GNPs in the WOF of the epoxy nanocomposite, due to the low agglomeration of EGNFs at this loading level.

Overall, the EGNF-reinforced epoxy nanocomposite at 0.25 wt.% loading and the GNP-reinforced epoxy nanocomposite at 1 wt.% loading demonstrated the best comprehensive mechanical properties in the respective group. In particular, the EGNF-reinforced epoxy nanocomposite at an ultra-low loading level (0.25 wt.%) not only outperformed the GNP counterpart and the neat epoxy for all of the mechanical properties, but also outperformed all of the GNP-reinforced epoxy nanocomposites in strength and stiffness, indicating the great potential of EGNFs in reinforcing epoxy-based nanocomposite materials. However, EGNFs have demonstrated much worse agglomeration than GNPs at higher loading, which might be due to fiber entanglement, and need to be further studied in future research.

### 3.3. Characterization of Surface-Modified EGNFs

**Figure 4:**
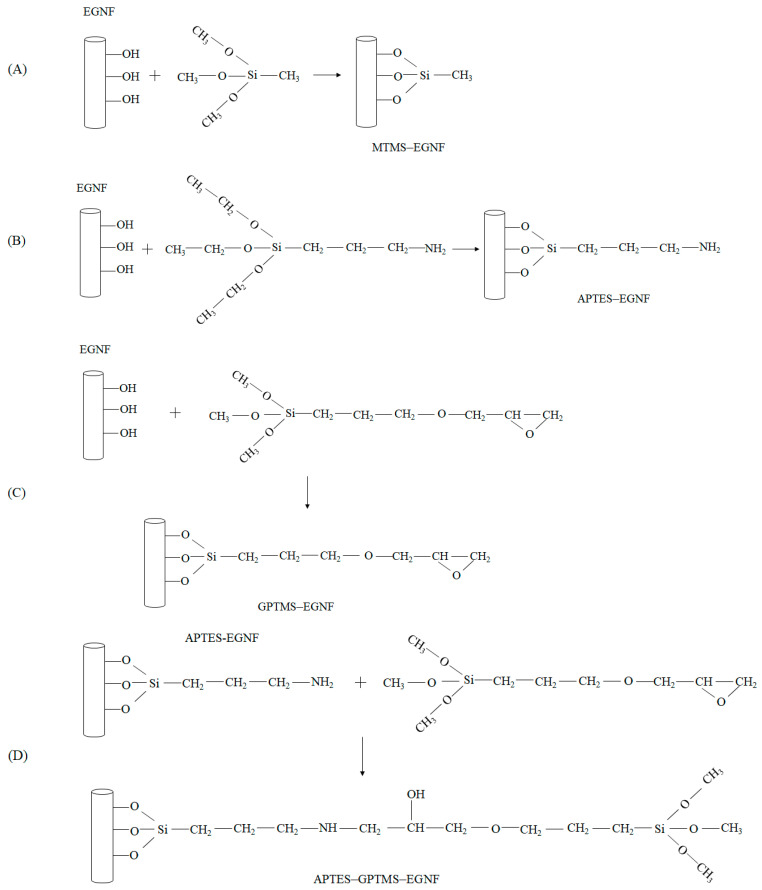
Schematic illustration of possible reactions between EGNFs and silane coupling agents: (**A**) MTMS; (**B**) APTES; (**C**) GPTMS; (**D**) APTES–GPTMS.

In order to study the effect of the surface-grafted silane chain length of EGNFs after silane treatment on the mechanical performance of the resultant epoxy nanocomposite, four types of silane coupling agents with increasing chain lengths, including MTMS, APTES, GPTMS, and APTES–GPTMS, were employed, respectively, to carry out the surface treatment of the EGNFs. According to the reaction mechanisms, as demonstrated in Figure 4, MTMS–EGNFs had the shortest grafted chain length on the EGNF surface, followed by APTES–EGNFs, GPTMS–EGNFs, and APTES–GPTMS–EGNFs.

**Figure 5:**
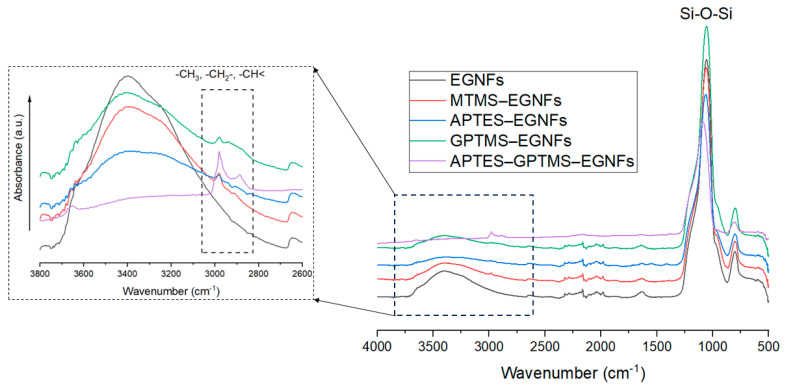
FTIR spectra of pristine EGNFs and surface-modified EGNFs with MTMS, APTES, GPTMS, and APTES–GPTMS.

FTIR was used to characterize the surface-modified EGNFs to confirm the graft of various silane molecules on the nanofiber surface. The FTIR spectra of the pristine EGNFs, MTMS–EGNFs, APTES–EGNFs, GPTMS–EGNFs, and APTES–GPTMS–EGNFs are compared in Figure 5. All the silane-treated EGNFs showed typical –CH_3_, –CH_2_–, and –CH < peaks in the range of 2800 cm^−1^–3000 cm^−1^, while the pristine EGNFs did not show any peaks in that range, indicating the successful graft of silane molecules on the surface of the EGNFs.

**Figure 6:**
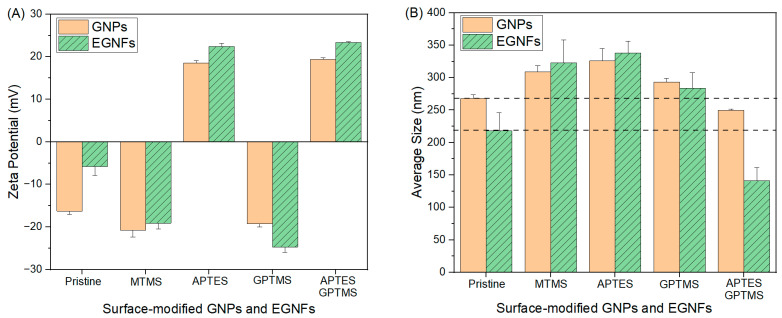
Average zeta potential (**A**), and average size (**B**) of pristine and surface-modified EGNFs and GNPs via DLS.

To investigate the physical stability of the silane-treated EGNFs, DLS was used to characterize the zeta potential and the average size of the surface-modified EGNFs (Figure 6). Surface modification with MTMS, APTES, GPTMS, and APTES–GPTMS caused a change in the average zeta potential of the pristine EGNFs, from −5.82 mV to −19.1 mV, +22.43 mV, −24.7 mV, and +23.35 mV, respectively. The average size of the pristine EGNFs via DLS was 218 nm. The utilization of MTMS, APTES, and GPTMS increased the average size of the resultant surface-modified EGNFs by 48%, 55%, and 30%, respectively, while APTES–GPTMS reduced the average size of the resultant surface-modified EGNFs by 35%. For comparison, the zeta potential and the average size of the pristine GNPs via DLS were −16 mV and 268 nm, respectively. The surface treatment of GNPs with MTMS, APTES, GPTMS, and APTES–GPTMS changed the zeta potential of the corresponding surface-modified GNPs to −20.75 mV, 18.55 mV, −19.2 mV, and 19.35 mV, respectively. The utilization of MTMS, APTES, GPTMS, and APTES–GPTMS slightly increased the average size of the GNPs by 15%, 22%, and 9%, respectively. Dual silane treatment with APTES–GPTMS reduced the GNP’s average size by 7%.

In DLS measurement, the particle size of sample is not measured directly, but is based on the movement of the particles. The obtained particle size is called the hydrodynamic diameter, which refers to the size of the smooth and spherical particles that diffuse at the same speed as the sample particles. EGNFs can be treated as nanorods [26] and their lengths affect their DLS hydrodynamic diameters. Therefore, the average sizes via DLS are only noted for comparison purposes. To acquire accurate size information for the surface-modified EGNFs, we used SEM image analysis (Table 1). We observed that the surface modification increased the average sizes of the EGNFs and GNPs, which could be ascribed to the silane self-condensation reaction and the subsequent connection of EGNFs and GNPs [27]. The dual silane-treated EGNFs and GNPs exhibited the smallest average sizes among all the surface-modified EGNFs and GNPs, respectively, indicating the least agglomeration caused by the two rounds of ultrasonication.

In terms of physical stability, as indicated by a higher absolute value of the zeta potential, as well as a smaller average size, the dual silane treatment involving APTES and GPTMS exhibited the most favorable overall outcome for the surface-modified EGNFs and GNPs. Grafting longer silane chains on the surfaces of EGNFs and GNPs tends to improve their stability with less agglomeration, which could be beneficial in further improving the mechanical properties of the corresponding EGNF- and GNP-reinforced epoxy nanocomposites.

### 3.4. Reinforcing Effect of the Surface-Modified EGNFs with Silane Coupling Agents Having Different Molecular Chain Lengths in Epoxy Nanocomposites

Based on the overall good comprehensive mechanical properties, 0.25 wt.% loading was used to study the effect of surface-modified EGNFs from silane treatment on the mechanical properties of the surface-modified EGNF-reinforced epoxy nanocomposites. For a similar reason, 1 wt.% loading was used to study the surface-modified GNP-reinforced epoxy nanocomposites, for comparison.

**Figure 7:**
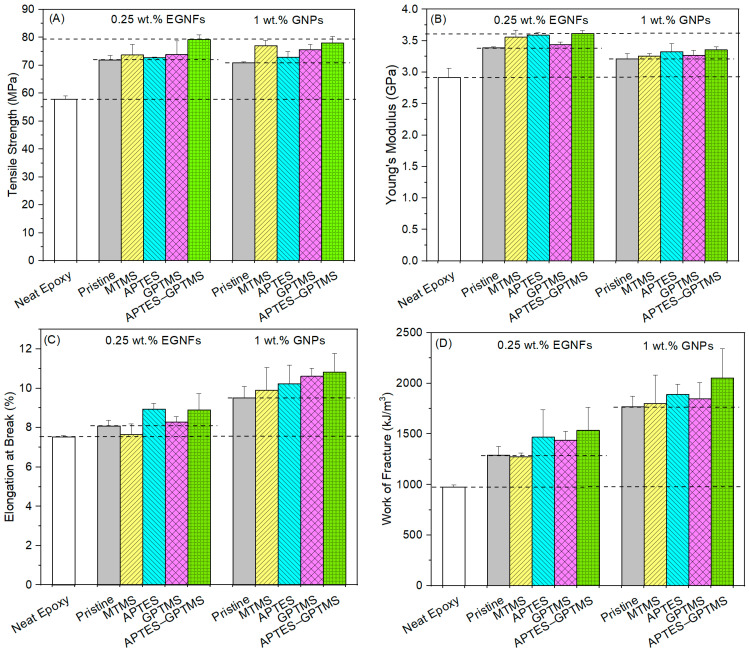
Tensile strength (**A**), Young’s modulus (**B**), elongation at break (**C**), and work of fracture (**D**) of neat epoxy (control sample) and epoxy nanocomposites reinforced with surface modified EGNFs at 0.25 wt.% and surface-modified GNPs at 1 wt.%.

We observed that, in most cases, the surface modification of EGNFs and GNPs with silane coupling agents showed significant improvement in all of the mechanical properties of the resultant epoxy nanocomposites with respect to those of the respective pristine EGNF and GNP counterparts (Figure 7). Among all the studied silane coupling agents, MTMS (which has the shortest molecular chain length) generally showed the least mechanical property improvement in the resultant MTMS–EGNF-reinforced epoxy nanocomposite, while APTES–GPTMS (which has the longest molecular chain length) showed the greatest mechanical property improvement in the resultant APTES–GPTMS–EGNF-reinforced epoxy nanocomposite. Among all the studied epoxy nanocomposites in this research, the APTES–GPTMS–EGNF-reinforced epoxy nanocomposite at 0.25 wt.% loading possessed the highest tensile strength of 79.3 MPa and the highest Young’s modulus of 3.62 GPa, which were improvements of 37% and 24%, respectively, with respect to those of the neat epoxy. The APTES–GPTMS–GNP-reinforced epoxy nanocomposite at 1 wt.% loading possessed the largest elongation at break of 10.8% and the largest WOF of 2052 kJ/m^3^, which were improvements of 44% and 110%, respectively, with respect to those of the neat epoxy.

The mechanical property improvement of the epoxy nanocomposites from the integration of surface-modified EGNFs (or GNPs) can first be attributed to the interaction between the surface-modified EGNFs (or GNPs, the filler) and the epoxy molecules (the matrix). MTMS–EGNFs have the shortest grafted molecular chain length on their surface and, thus, the weakest entanglement with surrounding epoxy molecules. APTES–GPTMS-EGNFs have the longest grafted molecular chain length on their surface and, thus, the strongest entanglement with surrounding epoxy molecules. APTES and GPTMS have molecular chain lengths between those of MTMS and APTES–GPTMS, while GPTMS possesses a slightly longer chain length than APTES. From the point of view of chain length and entanglement, GPTMS should outperform APTES in terms of reinforcing effect. However, APTES and GPTMS both contain reactive end groups, which could have chemical reactions with the epoxy molecules during the curing process. The amine functional groups of APTES could react with the epoxide functional groups of the epoxy resin during the mixing/curing process, while the epoxide functional groups of GPTMS could react with the epoxy curing agent in the process of curing, both of which complicate the APTES and GPTMS chain length effects. Overall, GPTMS and APTES showed close reinforcing effects on the mechanical properties of the surface-modified EGNF- (or GNP-) reinforced epoxy nanocomposites, while GPTMS demonstrated greater contribution to the strength of the resultant epoxy nanocomposites and APTES demonstrated greater contribution to the stiffness and toughness of the resultant epoxy nanocomposites.

Furthermore, the distribution of nanofillers in the epoxy matrix also played an important role. APTES–GPTMS–EGNFs had the smallest average size, indicating the least agglomeration and the most homogeneous distribution of APTES–GPTMS–EGNFs in the epoxy matrix compared to other silane-treated EGNFs. This was also applicable in the case of APTES–GPTMS–GNPs. Based on the longest graft chain length on the EGNF surface and the least agglomeration, the epoxy nanocomposite with APTES–GPTMS–EGNFs at 0.25 wt.% loading demonstrated the highest strength and the highest stiffness among all the studied epoxy nanocomposites (Table 2). The epoxy nanocomposite with APTES–GPTMS–GNPs at 1 wt.% loading showed the highest ductility and the highest toughness among all the studied epoxy nanocomposites, which is a benefit from the relatively high loading of GNPs. If we could significantly reduce the agglomeration of EGNFs at the same loading, the EGNFs would have performed better than the GNPs in the toughening effect, because of the “bridging” mechanism.

### 3.5. Fracture Surface of Surface-Modified EGNF-Reinforced Epoxy Nanocomposites and Reinforcing Mechanism

**Figure 8:**
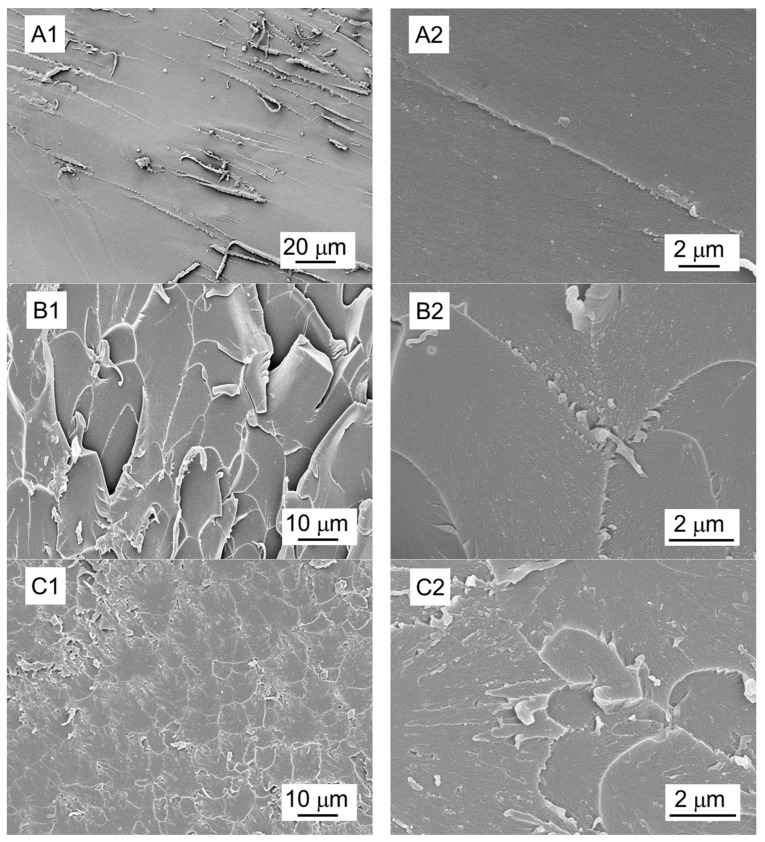

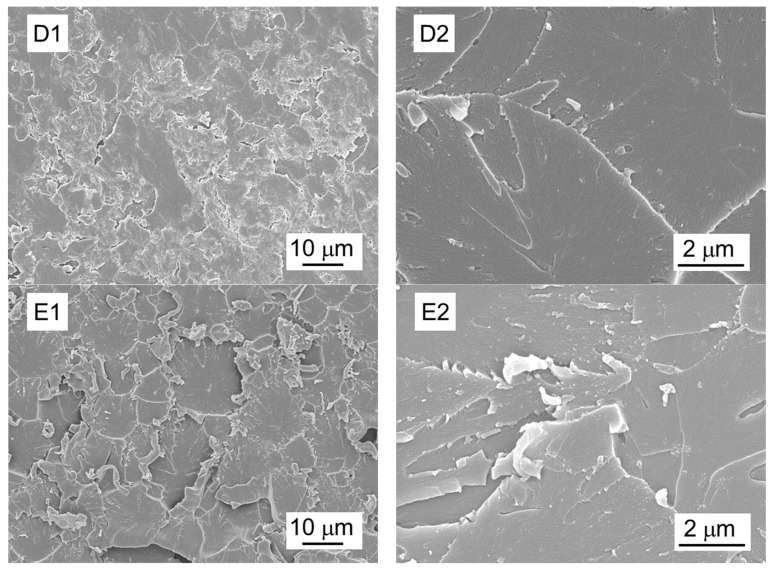
Representative morphology of fracture surfaces after tensile test: (**A**) neat epoxy; (**B**) epoxy nanocomposite with pristine EGNF at 0.25 wt.% loading; (**C**) epoxy nanocomposite with pristine GNP at 1 wt.% loading; (**D**) epoxy nanocomposite with APTES–GPTMS–EGNFs at 0.25 wt.% loading; (**E**) epoxy nanocomposite with APTES–GPTMS–GNPs at 1 wt.% loading. Label 1—low magnification; Label 2—high magnification.

To better understand the reinforcing effect imparted by the dual silane-treated EGNFs (APTES–GPTM–EGNFs) in the epoxy nanocomposite, fracture surfaces of the specimens of the APTES–GPTMS–EGNF-reinforced epoxy nanocomposite at 0.25 wt.% loading after the tensile test were examined by using SEM to link the interfacial bonding between the surface-modified EGNFs and the epoxy matrix (Figure 8). The fracture surfaces of the specimens of the APTES–GPTMS–GNP-reinforced epoxy nanocomposite at 1 wt.% loading after the tensile test were also examined by SEM, for comparison.

We observed that the neat epoxy (Figure 8A) exhibited a relatively smooth surface, with only a few discernible fracture lines that were oriented along the direction of crack growth, indicating low toughness. In comparison, as shown in Figure 8B,C, the epoxy nanocomposites with the pristine EGNFs and GNPs at 0.25 wt.% and 1 wt.% loadings, respectively, showed rough surfaces and multi-plane fracture lines, attributing to a tougher interface between the epoxy matrix and the nanofillers and, consequently, better mechanical properties than those of the neat epoxy. 

It is important to highlight that the EGNFs could exhibit the advantage of “bridging” effect, due to their large aspect ratios [21]. If a crack starts to form within the epoxy matrix under stress, EGNFs can stay intact across the crack surfaces, providing continuous resistance to the applied load until these fibers break completely. The epoxy nanocomposites with the dual silane treated nanofillers, i.e., APTES–GPTMS–EGNFs and APTES–GPTMS–GNPs, at 0.25 wt.% and 1 wt.% loading (Figure 8D,E), respectively, exhibited even rougher fracture surfaces than those with the pristine EGNFs and GNPs, which aligned with their superior mechanical properties. The detachment of the pristine EGNFs and GNPs from the epoxy matrix required energy, due to their large specific surface area as nanomaterials, and the detachment of APTES–GPTMS–EGNFs and APTES–GPTMS–GNPs from the epoxy matrix required even greater amounts of energy, due to the stronger interfacial adhesion achieved by the longer grafted molecular chains on the nanofiller surface and stronger entanglement with the epoxy matrix molecules therefrom. The “bridging” mechanism of EGNFs enabled the highest strength and stiffness, among all the studied epoxy nanocomposites, from the APTES–GPTMS–EGNF-reinforced epoxy nanocomposite at 0.25 wt.% loading, which even outperformed the 1 wt.% APTES–GPTMS–GNP-reinforced epoxy nanocomposite. However, the fracture surface of the APTES–GPTMS–EGNF-reinforced epoxy nanocomposite at 0.25 wt.% loading showed fewer fracture lines than that of the APTES–GPTMS–GNP-reinforced epoxy nanocomposite at 1 wt.% loading, suggesting lower toughness. This observation matched the mechanical test results and could be ascribed to the much smaller loading of EGNFs in the epoxy matrix than that of GNPs.

**Figure 9:**
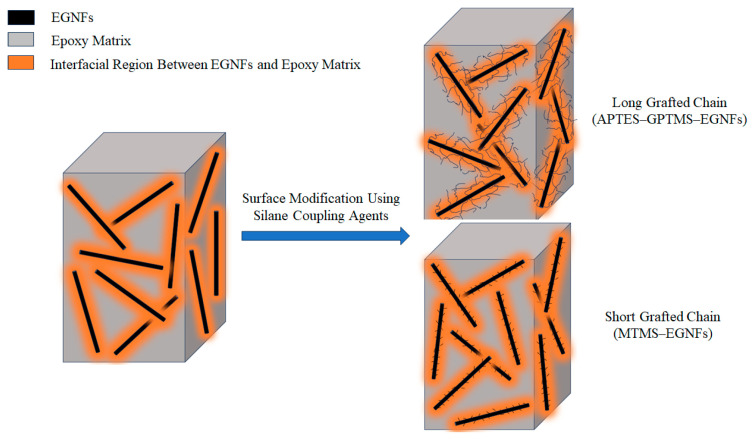
Schematic illustration of reinforcing mechanism using surface-modified EGNFs.

Overall, the surface-modified EGNFs provided great potential to reinforce epoxy resin. Longer grafted silane molecular chains on the surface of EGNFs could lead to stronger interfacial bonding between EGNFs and epoxy matrix, a result of stronger molecular entanglement (Figure 9). The whole mechanical performance of EGNF-reinforced epoxy nanocomposite is determined by comprehensive and balanced EGNF-epoxy interactions from the length of surface-grafted molecules, the “bridging” effect, and the agglomeration of EGNFs. In this research, the EGNFs exhibited adverse effects on reinforcement, due to the extent of agglomeration at loadings beyond 0.5 wt.%. Reducing the agglomeration of EGNFs is the key to future successful employment of EGNFs for highly efficient reinforcing purposes.

### 3.6. Thermal Stability of Surface-Modified EGNF-Reinforced Epoxy Nanocomposites

To assess the thermal stability of the pristine and surface-modified EGNF-reinforced epoxy nanocomposites, TGA was used to investigate decomposition temperatures of the EGNF-reinforced epoxy nanocomposites at 0.25 wt.% loading, corresponding to 10%, 20%, 60%, and 80% weight loss. The pristine and surface-modified GNP-reinforced epoxy nanocomposites at 1 wt.% loading were also examined, for comparison.

It was observed that the incorporation of the glass nanofillers in the epoxy resin could improve its thermal stability (Table 3). However, the surface modification of the EGNFs and the GNPs did not significantly change the thermal stability of the resultant epoxy nanocomposites.

## 4. Conclusions

In this research, glass nanofibers with an average diameter of ~90 nm were successfully prepared through electrospinning and, subsequently, surface-modified with a variety of silane coupling agents that had increasing chain lengths in the order of methyl trimethoxysilane (MTMS), (3-aminopropyl) triethoxysilane (APTES), (3-glycidyloxypropyl) trimethoxysilane (GPTMS), and dual silane APTES–GPTMS. The pristine and surface-modified electrospun glass nanofibers (EGNFs) were employed as reinforcing fillers in the epoxy resin at low loading levels (0.25 wt.%, 0.5 wt.%, and 1 wt.%), while a commercial product of glass nanoparticles (GNPs) was used as a control for comparison purposes. Compared to the neat epoxy, the incorporation of 0.25 wt.% pristine EGNFs increased the strength, stiffness, ductility, and toughness of the resultant epoxy nanocomposite by 24%, 16%, 7%, and 32%, respectively, and outperformed the pristine GNP counterpart. Further increasing the EGNF loading in the epoxy nanocomposite resulted in a significant decrease of its mechanical performance, due to EGNF agglomeration, which was much worse than that of the GNP counterparts at higher loadings. The surface modification of the EGNFs with silane coupling agents further improved the mechanical properties of the resultant epoxy nanocomposites. The surface-modified EGNFs with the longest grafted molecular chain on the surface via APTES–GPTMS treatment demonstrated the most effectiveness in mechanical property improvement of the resultant epoxy nanocomposite. The epoxy nanocomposites with 0.25 wt.% of APTES–GPTMS–EGNFs and 1 wt.% of APTES–GPTMS–GNPs showed the best comprehensive mechanical properties among all the studied epoxy nanocomposites. The former exhibited the highest strength and stiffness, i.e., tensile strength of 79.2 MPa and Young’s modulus of 3.62 GPa, improvements of 37% and 24%, respectively, with respect to those of the neat epoxy, while the latter showed the highest ductility and toughness, i.e., elongation at break of 10.8% and work of fracture of 2052 kJ/m^3^, improvements of 44% and 110%, respectively, with respect to those of the neat epoxy. The superior mechanical performance of epoxy nanocomposite with APTES–GPTMS–EGNFs at ultra-low loading (0.25 wt.%), i.e., 37%, 24%, 18%, 57% improvement in strength, stiffness, ductility, and toughness, respectively, with respect to those of the neat epoxy could be attributed to the longest grafted silane molecular chains on the EGNF surface and, consequently, the strongest interfacial adhesion, due to the greatest entanglement with epoxy matrix molecules, as well as the best distribution of the APTES–GPTMS–EGNFs in the epoxy matrix. However, a challenge of employing EGNFs in the epoxy matrix for further mechanical property improvement is their severe and increasing agglomeration, especially at higher loadings (>0.5 wt.%), which was a reason why the EGNFs demonstrated the best reinforcing effect at an ultra-low loading level (0.25 wt.%). 

The overall reinforcing performance of the surface-modified EGNFs is a result of comprehensive and balanced interactions with the epoxy matrix from the chain length of surface-grafted silane molecule, the “bridging” effect from the fiber shape, and the agglomeration of EGNFs. Reducing the agglomeration of EGNFs is the key to successful employment of EGNFs for reinforcing purposes. The scalability of EGNFs production, surface modification, and integration with epoxy resin is worthy of further investigation for future commercialization. This study pointed out directions for effectively designing and conducting surface modifications on nanofillers for high-performance polymer nanocomposite materials.

## Figures and Tables

**Table 1:** Average size (nm) of pristine and surface-modified EGNFs and GNPs from SEM image analysis.

Nanofiller	Pristine	MTMS	APTES	GPTMS	APTES–GPTMS
EGNFs	95 ± 35	150.43 ± 81.09	155.00 ± 63.89	143.50 ± 51.89	117.49 ± 63.27
GNPs	27.32 ± 8.35	49.384 ± 7.76	54.08 ± 16.08	53.15 ± 14.76	43.54 ± 10.53

**Table 2:** Percent improvement in the mechanical properties, including strength, stiffness, ductility, and toughness of the epoxy nanocomposites reinforced by pristine and surface-modified EGNFs and GNPs at loadings of 0.25 wt.% and 1 wt.%, respectively, with respect to the respective mechanical property of the neat epoxy.

Property	Nanofiller	Pristine	MTMS	APTES	GPTMS	APTES–GPTMS
Strength	EGNFs	24%	27%	26%	28%	37%
	GNPs	22%	33%	26%	31%	35%
Stiffness	EGNFs	16%	22%	23%	18%	24%
	GNPs	10%	12%	14%	12%	15%
Ductility	EGNFs	7%	1%	19%	10%	18%
	GNPs	26%	32%	36%	41%	44%
Toughness	EGNFs	32%	31%	51%	47%	57%
	GNPs	81%	84%	93%	89%	110%

**Table 3:** Thermal decomposition temperatures of the neat epoxy (control) and epoxy nanocomposites reinforced with pristine and surface-modified EGNFs at 0.25 wt.% loading and pristine and surface-modified GNPs at 1 wt.% loading.

Nanofiller	Thermal Decomposition Temperature (°C)			
	10% Weight Loss	20% Weight Loss	60% Weight Loss	80% Weight Loss
No nanofiller (neat epoxy)	377.63	386.02	410.38	451.08
EGNFs at 0.25 wt.% loading				
Pristine	381.59	390.65	414.85	473.7
MTMS	379.95	389.5	413.65	454.1
APTES	379.96	389.93	414.2	460.41
GPTMS	380.38	390.19	414.99	460.21
APTES–GPTMS	378.28	389.04	413.65	464.25
GNPs at 1 wt.% loading				
Pristine	381.46	390.6	415.29	462.89
MTMS	380.6	390.16	415.41	469
APTES	380.33	390.17	415.16	465.33
GPTMS	380.86	390.46	415.05	476.19
APTES–GPTMS	378.73	389.48	414.22	473.78

## Data Availability

The data presented in this study are available on request from the corresponding author.
